# Stent-Assisted Percutaneous Direct Endoscopic Necrosectomy in Necrotizing Pancreatitis With Multidrug-Resistant Infection and Portal Vein Thrombosis

**DOI:** 10.14309/crj.0000000000002118

**Published:** 2026-05-15

**Authors:** Ali Ghanem Al Masad, Samah Mosaad Soliman, Mana Saleh Al Qirad, Omar Reda Abdelmaksoud

**Affiliations:** 1 Department of Gastroenterology and Hepatology, Najran Health Cluster, King Khalid Hospital, Najran, Saudi Arabia; 2 Tanta University, Tanta, Egypt

**Keywords:** endoscopy, multidrug-resistant infections, necrotizing pancreatitis, walled-off pancreatic necrosis, portal vein thrombosis

## Abstract

Necrotizing pancreatitis with multidrug-resistant infection and portal vein thrombosis poses major therapeutic challenges. We report a critically ill 28-year-old man with walled-off pancreatic necrosis, portal and superior mesenteric vein thrombosis, and multidrug-resistant infection refractory to surgery, endoscopic retrograde cholangiopancreatography, and multiple percutaneous drainages. Percutaneous direct endoscopic necrosectomy using a mature surgical tract and stent-assisted working channel enabled 4-staged necrosectomy sessions with progressive source control, clinical recovery, and complete portal vein recanalization. This case highlights percutaneous direct endoscopic necrosectomy as a minimally invasive salvage option in complex necrotizing pancreatitis when surgery and transluminal approaches are high-risk or not feasible.

## INTRODUCTION

Necrotizing pancreatitis (NP) develops in ∼20% of acute pancreatitis cases and is associated with substantial morbidity and mortality. Infection of necrotic tissue is a major determinant of outcome and often necessitates intervention.^1–4^ Uncomplicated walled-off pancreatic necrosis is typically managed using endoscopic ultrasound (EUS)-guided transmural drainage with lumen-apposing metal stent placement, often followed by direct endoscopic necrosectomy when necessary. These minimally invasive step-up strategies have largely replaced open surgery and are associated with reduced morbidity and mortality.^5–7^ However, transluminal drainage requires close proximity of the collection to the gastric or duodenal lumen and may carry increased bleeding risk in the setting of portal hypertension or splanchnic vein thrombosis.^8,9^

We report a case of infected walled-off pancreatic necrosis in a patient with multidrug-resistant infection and concurrent portal and superior mesenteric vein thromboses in whom direct endoscopic necrosectomy (DEN) was unsafe due to unfavorable anatomy. A preexisting surgically created fistulous tract enabled percutaneous direct endoscopic necrosectomy (PDEN) as a salvage therapy. This case highlights the feasibility and safety of PDEN in a high-risk clinical context where conventional approaches were contraindicated.

## CASE REPORT

**Figure 1.**
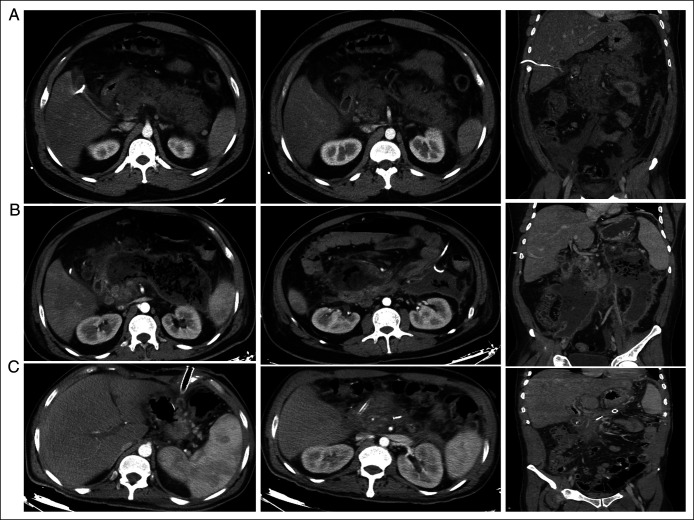
Contrast-enhanced CT imaging timeline. (A) Initial CT scan (day 1, May 29, 2025) demonstrating acute necrotizing pancreatitis with hypoenhancing pancreatic head necrosis and associated portal vein thrombosis, with extensive peripancreatic fluid extending into the paracolic gutters and pelvis. (B) Follow-up CT (June 19, 2025) showing development of a large organized walled-off necrosis at the pancreatic bed extending into both paracolic gutters despite prior drainage interventions. (C) Late follow-up CT (September 28, 2025) demonstrating near-complete resolution of the collection with residual mesenteric fat stranding and no recurrent fluid collection. Only uniform brightness/contrast adjustments were applied. No nonlinear or AI-based image modifications were used. CT, computed tomography.

A 28-year-old previously healthy man presented with severe epigastric pain, persistent vomiting, and new-onset jaundice. He was febrile (39°C), tachycardic (110 beats/min), and hypotensive (90/55 mm Hg), and had marked epigastric tenderness with guarding. Laboratory evaluation revealed leukocytosis (18,000/µL), hemoglobin 7.5 g/dL, platelets 110,000/µL, amylase 2,100 U/L, C-reactive protein 220 mg/L, creatinine 1.6 mg/dL, and cholestatic liver enzyme elevation. Bilirubin was 3.2 mg/dL (direct 1.2 mg/dL). Ultrasound demonstrated gallstones and a nondilated common bile duct. Contrast-enhanced computed tomography (CT) on day 2 showed acute biliary pancreatitis with >50% necrosis, retroperitoneal collections, and portal and superior mesenteric vein thromboses (Figure 1). Admission severity scores were BISAP 2 and APACHE-II 16.

The patient was admitted to the intensive care unit and treated with intravenous meropenem and aggressive fluid resuscitation. Therapeutic enoxaparin (80 mg every 12 hours) was initiated for portal and mesenteric vein thromboses as hemoglobin was stable and no active bleeding was present. Upper endoscopy excluded gastric varices. An interventional radiology-guided cholecystostomy was placed on day 10. He developed respiratory failure requiring mechanical ventilation and percutaneous tracheostomy on day 20.

Rising bilirubin prompted endoscopic retrograde cholangiopancreatography on day 26, during which sphincterotomy and clearance of multiple common bile duct stones were performed. Despite biliary decompression, the patient remained septic, and a laparoscopic necrosectomy with cholecystectomy and drain placement was performed on day 40. Drain amylase >1,000 U/L suggested pancreatic duct disruption, and a second endoscopic retrograde cholangiopancreatography with pancreatic duct stenting was performed on day 48. The pancreatic duct stent was maintained during recovery and later removed endoscopically after clinical stabilization and radiologic resolution of the collection. Serial CT scans on days 63 and 65 showed persistent large walled-off necrosis with gas (Figure 1). Interventional radiology placed additional 12 French percutaneous drains on day 66; cultures repeatedly grew multidrug-resistant *Klebsiella pneumoniae*, *Acinetobacter baumannii*, and vancomycin-resistant *Enterococcus faecium*, prompting escalation to meropenem, vancomycin, and colistin-based regimens.

Enteral feeding attempts via nasogastric and nasojejunal tubes were unsuccessful due to recurrent vomiting, and parenteral nutrition was initiated. After a multidisciplinary review, PDEN was selected as salvage therapy due to (1) an existing mature percutaneous surgical tract, (2) the cavity's remoteness from the gastric lumen, and (3) increased risk of transluminal access because of portal and mesenteric vein thromboses.

**Figure 2.**
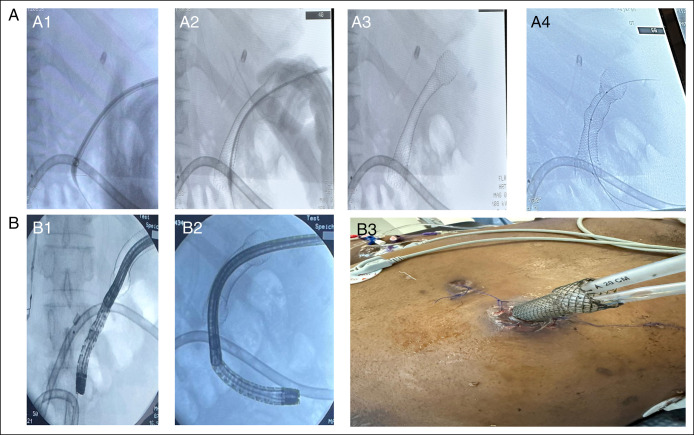
Fluoroscopic and external images demonstrating the stepwise technique of stent-assisted percutaneous direct endoscopic necrosectomy. (A1) Bougie dilation of the mature percutaneous fistulous tract over a guidewire. (A2) Deployment of a fully covered 123 mm × 18 mm esophageal self-expandable metal stent across the tract. (A3) Partial stent expansion with central luminal narrowing due to surrounding tissue compression. (A4) Controlled radial expansion balloon dilation performed to achieve full stent expansion and adequate luminal patency. (B1–B2) Fluoroscopic images demonstrating passage of the gastroscope into the right and left limbs of the necrotic cavity during the third session. (B3) External view of the stent exiting the skin with 2 surgical drains positioned through the stent to facilitate bilateral cavity drainage. Only uniform brightness/contrast adjustments were applied. No nonlinear or AI-based image modifications were used.

On day 80, a preexisting 12 French surgically placed drain provided access from the necrotic cavity to the skin. A guidewire was advanced through the drain under fluoroscopic guidance to confirm intraluminal positioning within the collection. The drain was then removed while maintaining wire access. The fistulous tract was sequentially dilated using bougie dilators up to 12 mm. A fully covered 123 mm × 18 mm esophageal self-expandable metal stent was deployed over the guidewire to establish a stable working channel. Following deployment, partial central compression of the stent was observed due to surrounding tissue pressure; therefore, controlled radial expansion balloon dilation to 15 mm was performed to ensure full expansion and allow safe passage of a 9.2 mm gastroscope into the necrotic cavity (Figure 2).

**Figure 3.**
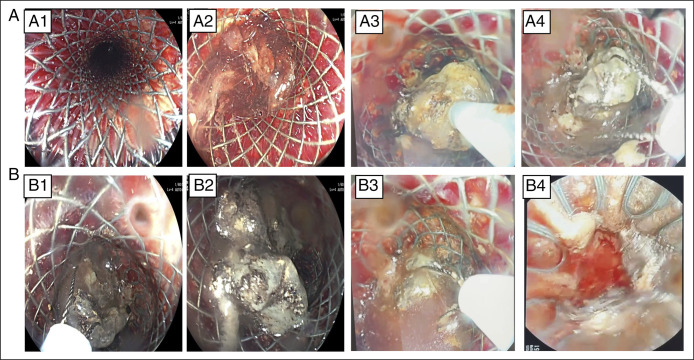
Endoscopic findings during the first session of stent-assisted percutaneous direct endoscopic necrosectomy. (A1) Endoscopic view through the lumen of the fully covered esophageal self-expandable metal stent, demonstrating a patent working channel. (A2) Visualization of necrotic debris and turbid fluid at the distal end of the stent within the walled-off necrosis cavity. (A3) Removal of necrotic tissue using a snare. (A4, B1, B3) Extraction of necrotic debris using a Roth net. (B2) Large fragments of devitalized pancreatic necrosis within the cavity. (B4) Postdebridement view showing partial clearance of the necrotic material after completion of the first session. Only uniform brightness/contrast adjustments were applied. No nonlinear or AI-based image modifications were used.

**Figure 4.**
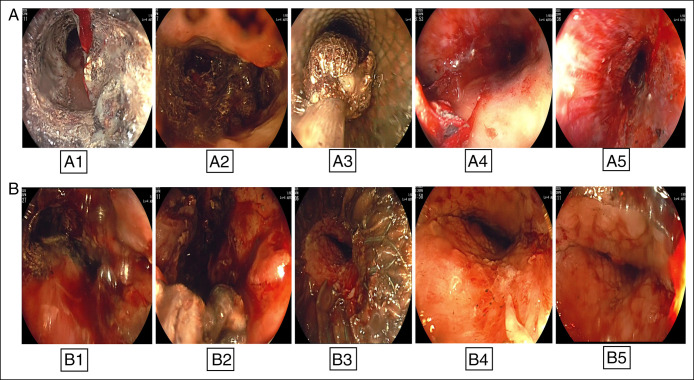
Endoscopic views during the third (A) and fourth (B) sessions of stent-assisted percutaneous direct endoscopic necrosectomy. (A1–A2) Persistent necrotic pancreatic debris within the cavity during the third session. (A3) Removal of necrotic tissue using a snare. (A4–A5) Right limb of the cavity following debridement, demonstrating progressive clearance of the necrotic material. (B1–B2) Residual necrotic debris within the left limb of the cavity during the fourth session. (B3) Distal end of the stent with entrance to the cavity following substantial debridement. (B4) Entrance and transverse portion of the cavity showing granulation tissue after clearance. (B5) Complete clearance of the left limb with healthy granulation tissue formation. Only uniform brightness/contrast adjustments were applied. No nonlinear or AI-based image modifications were used.

Four PDEN sessions were performed at 5-day intervals. Necrotic debris was removed using snares, Roth nets, rat-tooth forceps, and saline irrigation, with 100–300 mL extracted per session (first session, see Figure 3). Progressive cavity clearance was achieved during the third and fourth sessions, with visualization of healthy granulation tissue and near-complete removal of necrotic debris (Figure 4). The esophageal stent was left in situ between sessions to maintain tract patency and allow repeat endoscopic access. A surgical drain was readvanced through the lumen of the stent into the necrotic cavity to facilitate interim drainage. The external openings of both the stent and the drain were covered with a stoma appliance to permit controlled passive drainage and protect the surrounding skin; no active suction was applied directly to the stent. Drains were exchanged after each procedure, with interim saline irrigation as needed (Figure 2). Anticoagulation management followed guideline-supported practice: The morning enoxaparin dose was withheld and resumed the same evening after confirmation of hemostasis.^10^ No bleeding, perforation, or stent migration occurred.

Clinical improvement was progressive. Fever resolved, vasopressors were discontinued, and leukocytosis normalized. Cultures became sterile after the third session. Following completion of the fourth necrosectomy session, the esophageal stent was exchanged for a smaller-diameter stent to facilitate gradual tract downsizing. After sustained clinical improvement, the stent was removed endoscopically. The external fistulous opening was managed conservatively with sterile dressings and closed spontaneously by secondary intention over approximately 3 weeks. No persistent external pancreatic fistula was observed.

Three-month CT showed reduction of the collection from 14 cm × 10 cm to 3 cm × 2 cm (Figure 1), with complete portal vein recanalization. At 4 months, the patient remained clinically stable, was tolerating an oral diet, and had been discharged home following transfer from the intensive care unit to the general ward. At 6-month follow-up, he remained asymptomatic with no recurrence of pancreatic collections (Table 1).

**Table 1.** Timeline of clinical course, interventions, and PDEN response

Day	Intervention	Key laboratory values/physiology	Microbiology	Imaging	Complications	Outcome
1	ICU admission	WBC 18,000/µL; CRP 220 mg/L; temp 39°C; norepinephrine 0.2 μg/kg/min; intubated	Pending	CT: >50% necrosis; PVT + SMV thrombosis	—	Stabilized, ventilated
26	ERCP #1 (sphincterotomy, stone clearance)	Bilirubin improving	Bile clear	Dilated CBD	—	Duct cleared
40	Laparoscopic necrosectomy + cholecystectomy	Persistent sepsis	MDR later	Large WOPN	—	Drains placed
48	ERCP #2 (pancreatic duct stent)	High-drain amylase	MDR persists	WOPN unchanged	—	PD stent placed
66	IR drainages	Elevated CRP/WBC	MDR confirmed	Loculated abscesses	—	Minimal response
80	PDEN #1	WBC 14,000/µL; CRP 160 mg/L; temp 38°C; norepinephrine 0.12 μg/kg/min	Positive	Cavity with debris	—	100 mL removed
85	PDEN #2	WBC 12,000/µL; CRP 120 mg/L; temp 37.8°C; norepinephrine 0.08 μg/kg/min	Positive	Persistent debris	—	100 mL removed
90	PDEN #3	WBC 10,000/µL; CRP 80 mg/L; temp 37.5°C; norepinephrine 0.05 μg/kg/min	Improving	Debris decreasing	—	300 mL removed
95	PDEN #4	WBC 8,000/µL; CRP 40 mg/L; temp 37°C; off vasopressors	Sterile	Granulation tissue	—	300 mL removed
3 mo	CT follow-up	WBC 7,000/µL; CRP <5 mg/L; afebrile	Sterile	WOPN 3 cm × 2 cm; portal vein recanalized	—	Stable, off support

CBD, common bile duct; CRP, C-reactive protein; CT, computed tomography; ERCP, endoscopic retrograde cholangiopancreatography; ICU, intensive care unit; MDR, multidrug-resistant; PD, pancreatic duct; PDEN, percutaneous direct endoscopic necrosectomy; PVT, portal vein thrombosis; SMV, superior mesenteric vein; WBC, white blood cell; WOPN, walled-off pancreatic necrosis.

## DISCUSSION

Severe NP complicated by portal and superior mesenteric vein thrombosis and multidrug-resistant infection poses significant therapeutic challenges. Achieving safe and effective source control is particularly difficult because vascular thrombosis increases the risk of catastrophic bleeding during invasive procedures.^11^

Randomized trials support the step-up approach and DEN for walled-off necrosis, demonstrating reduced systemic complications, mortality, and hospital stay compared with open surgery.^1–4^ However, DEN requires close proximity of the collection to the gastric or duodenal lumen and carries bleeding risk, especially in patients with portal hypertension or thrombosis.^8,9^ Evidence for PDEN remains limited to small series and case reports,^11–17^ and its role in patients under therapeutic anticoagulation has not been well described.

Interfacility transfer to a center with EUS-guided necrosectomy capability was considered; however, the patient remained hemodynamically unstable, mechanically ventilated, and intensive care unit-dependent during persistent sepsis. Given the high-risk clinical status and multiple existing drainage interventions, transfer was deemed unsafe and impractical. In this case, PDEN was favored for several reasons: (1) a mature surgical tract provided secure access; (2) the necrotic cavity was anatomically distant from the gastric lumen, making transluminal DEN unsafe; (3) prior surgery and multiple percutaneous interventions had failed to achieve source control; and (4) lack of EUS availability at the treating center. PDEN enabled stepwise debridement while preserving viable tissue and promoting granulation.

Therapeutic anticoagulation posed a procedural challenge. Withholding a single low-molecular-weight heparin dose and resuming after hemostasis align with guideline recommendations and minimized bleeding risk.^7^ No hemorrhagic events occurred.

Multidrug-resistant organisms prolonged sepsis despite maximal medical therapy. Definitive improvement coincided with mechanical source control, reinforcing that antimicrobial therapy alone is insufficient in infected necrosis.^18,19^ Nutritional management attempted early enteral feeding, but intolerance required temporary parenteral support until granulation and clinical stabilization allowed reintroduction, consistent with best practices.^20^

Resource demands—fluoroscopy, anesthesia support, and metallic stents—may limit widespread adoption. PDEN may be considered in carefully selected patients with a mature percutaneous tract, failure of conventional step-up strategies, collections anatomically remote from the gastric or duodenal lumen, or when EUS-guided access is contraindicated or unavailable. In selected cases with multiple collections, PDEN may provide targeted access if safe percutaneous routes exist. However, the technique requires advanced endoscopic expertise and multidisciplinary coordination.

Controlled external drainage via a mature tract reduces intracavitary pressure and facilitates evacuation of the infected material. Strict aseptic wound care was maintained, and no secondary soft tissue infection or cellulitis occurred. The tract closed spontaneously without complication.

Limitations include off-label stent use, absence of anti-Xa monitoring, and limited long-term follow-up.

In conclusion, PDEN provided effective, minimally invasive source control in a critically ill patient with multidrug-resistant infected NP complicated by portal and superior mesenteric vein thrombosis. The case illustrates that PDEN can serve as a salvage option when conventional surgical or transluminal approaches are contraindicated. Successful outcomes depend on individualized, multidisciplinary management with careful attention to anticoagulation and infection control. These findings are hypothesis-generating, and prospective studies are needed to define indications, safety, and long-term outcomes of PDEN.

## DISCLOSURES

Author contributions: AGA Masad: conceptualization and methodology, primary endoscopist and major role in endoscopy. AGA Masad, OR Abdelmaksoud, and MSA Qirad: endoscopic procedure. OR Abdelmaksoud: writing of original draft. All authors reviewed and editing the manuscript. AGA Masad and OR Abdelmaksoud provided supervision. AG Al Masad and OR Abdelmaksoud are the article guarantors.

Acknowledgments: We thank Dr. Mohamed Erfan (Consultant of General Surgery), the General Surgery and Anesthesia teams at King Khalid Hospital, and the endoscopy nursing staff for their support. No external writing assistance was used.

Financial disclosure: None to report.

Informed consent was obtained for this case report.

AI/LLM disclosure: ChatGPT (OpenAI) was used solely for language editing and formatting guidance. All clinical content was written, reviewed, and verified by the authors, who take full responsibility for its accuracy and integrity.

## ABBREVIATIONS:

CBD: common bile duct
CRP: C-reactive protein
CT: computed tomography
DEN: direct endoscopic necrosectomy
ERCP: endoscopic retrograde cholangiopancreatography
EUS: endoscopic ultrasound
ICU: intensive care unit
IR: interventional radiology
MDR: multidrug-resistant
NP: necrotizing pancreatitis
PDEN: percutaneous direct endoscopic necrosectomy
SMV: superior mesenteric vein
WOPN: walled-off pancreatic necrosis
